# Letter from Sir Henry Burdett, K.C.B.

**Published:** 1918-07-27

**Authors:** Henry C. Burdett

**Affiliations:** The Lodge, 13 Porchester Square, W. 2


### THE LONDON HOSPITAL NURSES.

### A Race between Statesmanship and Economics.

There is a quiet expectation abroad in the nursing world that the claims of the two year olds ought to win out. Economics won't be permitted to halve the salaries of any workers amongst the sick, after the war. The London Hospital, at the moment, has a unique opening to show and lead the way. If this splendid opportunity is allowed to pass, unity, the keynote to speedy justice for the nurse and her highest interests, may be delayed, temporarily, but the speed will quicken again to victory, through other Training Schools, as history demonstrates.

THE following letters appeared in *The Times* of July 15, 16, and 17 respectively. Previous correspondence on this subject was published in our issues of July 13, p. 333, and July 20, p. 353.

### Letter from Sir Henry Burdett, K.C.B.

SIR,—Viscount Knutsford's letter in *The Times* of the 12th instant has caused universal regret throughout the nursing world. The full publicity given in *The Times* of the 5th instant to the discussion on the above subject in the House of Commons on the 4th instant, and the action of the Speaker, afford the London Hospital a golden opportunity, by a slight technical concession in procedure, to close the ranks of the training schools and do justice to probationer nurses of the best, who wish to qualify themselves for advancement in their profession.

No probationer in the present day with a two-years' certificate of training is fully equipped to discharge adequately all the duties of a trained nurse. The practice at the London Hospital is to provide that a nurse with a two-years' certificate employed on the private staff shall only be sent to cases of which she has had experience. Colonel Maurice's letter exhibited the injustice on the part of a training school to make the instruction of the junior members of the nursing staff secondary to the demands on its private staff. The first duty of a training school is to the probationer who enters for full training. Three years' consecutive training is the standard now universally enforced throughout the nursing world, with the practical exception of the London Hospital.

The nation and Parliament are united in their desire to encourage and secure justice for the trained nurse. Lord Knutsford yields to no one in the interest he personally takes in nurses, and we all hoped, seeing that the London Hospital has issued, and does issue, a three-years' certificate of training, the whole of which period has been spent in practical work in the London Hospital, that he would waive the technicality and announce that in future probationers would be accepted for three years' training, spent in practical work in that institution, and so close the ranks.

The time has come to divorce the training of nurses from its present economic aspects, and to make the welfare and future of every probationer the first consideration, not only in justice to herself, but in justice to the public, in whose service she proposes to spend the best years of her life. Unity has the maximum of importance for British nurses to-day, and I do but voice the wish of the nursing profession when, greatly daring, I venture to ask space in *The Times* to make this appeal to the Chairman of the London Hospital.—I am, yours faithfully,

HENRY C. BURDETT.

The Lodge, 13 Porchester Square, W. 2, July 13.

